# Evolutionary biogeography of the centipede genus *Ethmostigmus* from Peninsular India: testing an ancient vicariance hypothesis for Old World tropical diversity

**DOI:** 10.1186/s12862-019-1367-6

**Published:** 2019-02-01

**Authors:** Jahnavi Joshi, Gregory D. Edgecombe

**Affiliations:** The Natural History Museum, Cromwell Road, London, SW7 5BD UK

**Keywords:** Gondwana biogeography, Western and eastern Ghats, Scolopendridae, Palaeoclimate

## Abstract

**Background:**

Understanding the relative influence of vicariance and dispersal in shaping Old World tropical biodiversity remains a challenge. We aimed to infer the roles of these alternative biogeographic processes using a species time-tree for the centipede genus *Ethmostigmus* from the Old World tropics. Additionally, we explored fine-scale biogeographic patterns for an endemic radiation of *Ethmostigmus* from the peninsular Indian Plate (PIP), an area with complex geological and climatic history.

**Results:**

Divergence time estimates suggest that *Ethmostigmus* began diversifying in the Early Cretaceous, 125.4 (± 25) million years ago (Ma), its early biogeographic history shaped by vicariance. Members of *Ethmostigmus* in PIP form a monophyletic group that underwent endemic radiation in the Late Cretaceous, 100 (± 25) Ma. In contrast, a new species of *Ethmostigmus* from north-east India formed a clade with African/Australian species. Fine-scale biogeographic analyses in PIP predict that Indian *Ethmostigmus* had an ancestor in southern-central parts of the Western Ghats. This was followed by four independent dispersal events from the southern-central Western Ghats to the Eastern Ghats, and between different parts of the Western Ghats in the Cenozoic.

**Conclusions:**

Our results are consistent with Gondwanan break-up driving the early evolutionary history of the genus *Ethmostigmus*. Multiple dispersal events coinciding with geo-climatic events throughout the Cenozoic shaped diversification in PIP. *Ethmostigmus* species in PIP are restricted to wet forests and have retained that niche throughout their diversification.

**Electronic supplementary material:**

The online version of this article (10.1186/s12862-019-1367-6) contains supplementary material, which is available to authorized users.

## Background

**Fig. 1:**
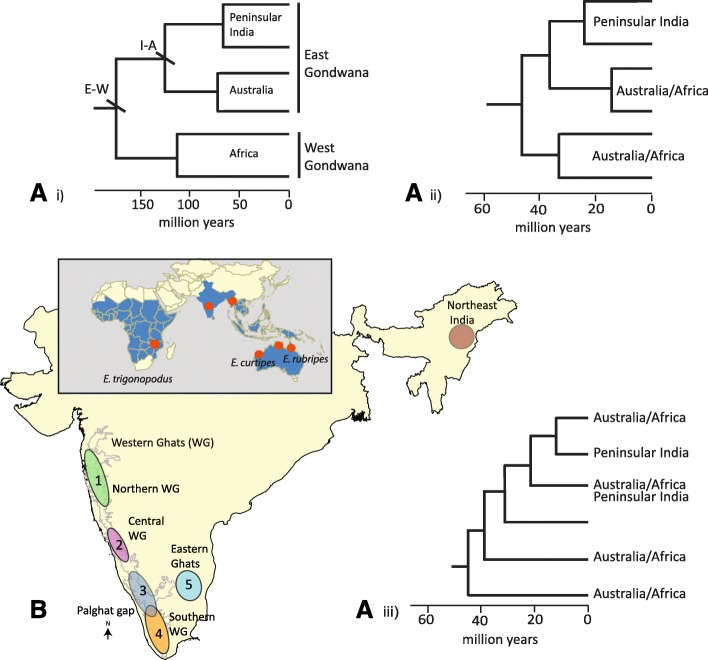
**a** Phylogenetic predictions for Gondwanan vicariance and long-distance hypotheses for Old World tropical diversity: i) Gondwanan ancient vicariance; the first vicariance event when East Gondwana (India-Madagascar-Australia) separated from Africa-South America (E-W); another vicariance event separating Australia from India-Madagascar (I-A); ii) long-distance dispersal across continents, leading to distinct lineages; and iii) multiple dispersal/vicariance scenario in which each landmass was colonised multiple times with younger divergence dates; **b**) Geographic distribution of the genus *Ethmostigmus* in the Old World tropics (red dots indicate where *Ethmostigmus* species have been sampled for biogeographic analyses) and approximate species distribution ranges in the peninsular Indian Plate 1. *E. sahyadrensis*, 2. *E. praveeni*, 3. *E. coonooranus*, 4. *E. agasthyamalaiensis,* and 5. *E. tristis* (based on Joshi and Edgecombe, 2018)

Understanding distribution patterns and processes that influence Old World tropical biodiversity is a longstanding question. The respective roles of biogeographic processes - vicariance and dispersal events - have repeatedly been debated, along with other ecological and evolutionary processes, to explain the origins and distribution of Old World tropical diversity [1, 2]. The Gondwanan ancient vicariance hypothesis predicts that each of the areas in the Old World tropics (e.g. the African subcontinent, Australia, and peninsular India) will have sister taxa with deep and old divergence dates, the latter being consistent with the age of separation of the Gondwanan fragments (Fig. 1A i). An alternate biogeographic hypothesis posits that either long-distance dispersal events likewise resulted in sister and distinct lineages on each of the landmasses, but their divergence dates would not coincide with the breakup of Gondwana (Fig. 1A ii) or there could have been multiple dispersal/vicariance events, resulting in non-monophyletic taxa, and again with divergence dates younger than Gondwana breakup (Fig. 1A iii) [3–6]. In recent years, it has been possible to evaluate these alternative hypotheses given access to time-calibrated molecular phylogenies and development of quantitative biogeographic methods for reconstructing ancestral areas for clades.

Historical biogeography of multiple taxa from West Gondwana (Africa and South America) and parts of East Gondwana (Australia and New Zealand) is well studied. Gondwana breakup has been invoked in some cases (e.g., geckos – [7]; plants – [8]; birds – [9]; beetles – [10]), whereas many others were explained by long-distance dispersal (e.g., starfish – [11]; several plant clades – [12–16]). Interestingly, in some taxa variance shaped the early history of lineages but was then followed by long-distance dispersal events or interchange of biotas across land bridges (beetles – [17]; plants – [18, 19]; birds – [9]). However, little is known about taxa with broader Old World tropical distributions, including parts of both East and West Gondwana, i.e., India, Madagascar, Australia, and Africa. Many ancient lineages with Old World tropical distributions that could potentially be explained by Gondwanan vicariance remain to be explored using time-calibrated phylogenies with fossils in a quantitative biogeographic framework. Consequently, understanding the relative contribution of ancient vicariance and long-distance dispersal remains a challenge in accounting for the origins of Old World tropical diversity.

Given limited dispersal ability of centipedes and the ancient age indicated by their fossil record [20], they are a suitable candidate for assessing the ancient vicariance and long-distance dispersal scenarios. Among scolopendrid centipedes, the genus *Ethmostigmus* [21] is an ideal example given its predominantly Old World tropical distribution. It includes 19 extant species, found across much of Africa, in India and Sri Lanka, Southeast Asia, the East Indies, Australia, Melanesia and Polynesia [22–27]. Earlier molecular phylogenetic studies on the family Scolopendridae suggest that *Ethmostigmus* began diversifying in the Early Cretaceous [28], therefore it allows an assessment of the roles of ancient vicariance and long-distance dispersal on its distribution. Detailed taxonomic studies have been conducted on *Ethmostigmus* from Australia (5 species, [24]) and peninsular India (5 species, [23]), describing endemic radiations. The distribution records for Africa [27] and Southeast Asia and Melanesia [25, 26] based on museum collections have also been assessed. Therefore, the current distribution and diversity patterns of the genus *Ethmostigmus* are reliable across the Old World tropics (Fig. 1B). However, as mentioned earlier, testing biogeographic hypotheses is contingent upon access to well-resolved phylogenetic relationships among species and age estimates for divergences. Therefore, in this study, we reconstruct a species time-tree including 9 of the 19 species of *Ethmostigmus* using three fossil calibrations in a Bayesian framework. These species represent four main areas, namely peninsular India, Australia, Africa and Southeast Asia (North-east India), to evaluate the aforementioned biogeographic hypotheses.

In the context of tropical Asia, the peninsular Indian Plate (PIP) is geologically distinct, being part of the Gondwanan supercontinent around 200 million years ago [29]. The region harbours a global biodiversity hotspot, the Western Ghats, on the western escarpment on the west coast. Dry/semi-arid scrub and grassland in the peninsula comprises a broken, much-isolated chain of low mountains with patchy wet forests on top on the eastern escarpment, the Eastern Ghats (Fig. 1B). In addition to continental breakup and movements, PIP also experienced prolonged and extensive volcanism around 65 Ma ago and dynamic palaeoclimatic shifts in the Cenozoic [30–33]. A few recent studies have explored the role of palaeoclimate on diversification and biogeography of small vertebrates in PIP. Different palaeoclimate events have been invoked, including global C4 grassland expansion, seasonality, the onset of the Asian monsoon, wet and dry habitat expansion, and fragmentation in the Paleogene or Miocene to explain biogeography and diversification among these taxa. Evolutionary history of many of these groups (especially in lizards and skinks) was shaped by the into-India dispersal event after the Indian Plate collided with Asia either from Southeast Asia, the Palearctic, or Sub-Saharan Africa origins [34–40]. Exceptions to this pattern include caecilians [41] and snakes [42], which have ancient Gondwanan origins. However, understanding of biogeography and diversification of ancient terrestrial invertebrates in PIP has been very limited, an exception being a study on the scolopendrid centipede genus *Digitipes* from the Western Ghats [32]. Given this, we were interested in exploring evolutionary biogeography of the endemic *Ethmostigmus* species in PIP. Specifically, we focused on the following questions: 1) When did *Ethmostigmus* start diversifying in PIP? Was the biogeographic history shaped by Gondwanan breakup or one/more long-distance dispersal events in PIP? 2) Given that *Ethmostigmus* species in PIP are largely restricted to the wet forests, were they influenced by Paleogene or Miocene or more recent Pleistocene climatic fluctuations and associated expansion and fragmentation of the forests?

## Methods

### Molecular phylogeny and divergence time estimation

Three centipede fossils can be used to calibrate the phylogeny for divergence time estimation. Among these, only one named fossil species has been identified as a member of the family Scolopendridae but none have yet been recognised as the subfamily Otostigminae or the genus *Ethmostigmus*. The oldest named fossil available for Scolopendridae is *Cratoraricrus oberlii*, from the Lower Cretaceous (Aptian) Crato Formation of Brazil [43]. We used this as crown group Scolopendridae based on extended sternal paramedian sutures indicative of the subfamily Scolopendrinae [20]. A minimum age of 113 ± 0.4 Ma dates the Aptian/Albian boundary, an Aptian age for the Nova Olinda Member of the Crato Formation being constrained by palynomorphs [44]. The second calibration fossil is *Devonobius delta*, from the upper part of the Panther Mountain Formation at the Middle Devonian Gilboa locality, Schoharie County, New York State, USA. Phylogenetic analysis resolves it as sister group of Epimorpha ([45], Fig. S3, therein) and thus it constrains crown-group Pleurostigmophora (Craterostigmomorpha, Geophilomorpha and Scolopendromorpha) with a minimum age of 382.7 ± 0.1 Ma. This dates the Givetian/Frasnian boundary, palynomorphs from the Panther Mountain Formation being of Givetian age [41]. Earlier phylogenetic analyses [46, 47] are also consistent with a crown-group Pleurostigmophora assignment, even if an alternative sister group relationship with Craterostigmomorpha is advocated. The third fossil calibration is provided by *Mazoscolopendra richardsoni*, from Pennsylvanian deposits of Mazon Creek, Illinois, USA. This total-group scolopendromorph (phylogeny from ([45], Fig. S3, therein) constrains crown group Epimorpha, with a minimum age of 307 ± 0.1 Ma. This dates the Francis Creek Shale Member of the Carbondale Formation to Westphalian D, or latest Moscovian in the global timescale, the absolute date being that of the Moscovian/Kasimovian boundary [41].

In recent literature, several studies have argued for the use of multiple fossil calibrations in divergence date estimation and have highlighted the limitations of secondary calibrations [48]. Hence, to obtain robust divergence estimates in which all three calibrations can be used, a larger dataset than the focus taxa of the current study was compiled. DNA sequences were assembled for the family Scolopendridae, exemplars of the families Cryptoptidae, Plutoniumidae and Scolopocryptopidae (also belonging to Scolopendromorpha), exemplars of several families from the sister order Geophilomorpha, and more distantly allied Craterostigmomorpha to constrain divergence estimates for *Ethmostigmus*. Species from the centipede orders Lithobiomorpha and Scutigeromorpha served as outgroups. A dataset of 368 specimens for three markers, two mtDNA genes (16S ribosomal RNA – 16S rRNA and cytochrome *c* oxidase subunit I - COI) and one nuclear gene (28S ribosomal RNA – 28S rRNA), was compiled for published studies [23, 28, 46, 49–60] (See SI Table1 for details). Additionally, we also sequenced two individuals of a new *Ethmostigmus* species from northeast India. DNA extraction, PCR purification and DNA sequencing protocols were as described in earlier work on the family Scolopendridae [23, 28]. Chromas Lite 2.1.1. was used to view and edit DNA sequences. Individual gene alignments were done using CLUSTAL W and MAFT [61, 62] in Geneious 8.1.4 (https://www.geneious.com/; [accessed on 6th Sept 2018]).

We used a species tree to account for lineage or species discord arising from random gene-lineage coalescence. The species tree reconstruction was carried out in starBEAST2 using a multi-species coalescent (MSC) framework for the family Scolopendridae and was implemented in BEAST 2.5 under a random relaxed clock with an exponential distribution [63]. PartitionFinder 2 was implemented to select a nucleotide substitute model and best partition scheme (gene partitions) using the greedy algorithm, and the Akaike Information Criterion (AIC) to compare the fit of different models. The best scheme selected was a three-gene partition (COI, 16S and 28S) with a GTR + I + G substitution model. Therefore, GTR + I + G was parameterized and set as a substitution model for all three partitions. For estimates of gene trees and clock models, the mitochondrial loci COI and 16S were treated as a single locus independent of the nuclear gene 28S. The ploidy assignments for mtDNA genes were set to haploid and 28S rRNA to diploid. For a speciation prior, a Yule speciation model was selected. The population model was set as “Analytical Population Size Integration”. BEAST was run for 5 × 10^9^ generations on the CIPRES server [64] in which parameters and trees were stored every 5 × 10^3^ generations, and convergence was determined by assessing stationary distribution using the program Tracer (v1.6) as well as by evaluating the effective sample sizes (> 200). Species Trees were resampled in LogCombiner v2.4.7 with a frequency of 2 × 10^5^ and 25% burn-in. A consensus tree was then obtained in TreeAnnotator (v1.8) and was visualized with FigTree (v1.4.3). The divergence date estimation was carried out using the three node calibrations described above with lognormal distribution priors.

### Biogeographic analyses

Historical biogeographic analyses for *Ethmostigmus* were performed using the R package “BioGeography with Bayesian (and likelihood) Evolutionary Analysis of RangeS (BioGeoBEARS)” [65]. This compares alternative biogeographic models and approaches in a hypothesis-testing framework using maximum likelihood, with a goal of testing the relative roles of ancient vicariance and long-distance dispersal. Specifically, we performed dispersal-vicariance analysis (DIVA) and dispersal-extinction-cladogenesis (DEC) in which probabilistic inference of ancestral geographic ranges was evaluated in a maximum likelihood framework. The ancestral area reconstruction was carried out at two scales, at the broad-scale across the Old World tropics and then to examine finer patterns within peninsular India. Each species was assigned to the following biogeographic regions based on known distributional ranges: Peninsular Indian Plate (A); Continental Asia (including northeast India, which is biogeographically closer to mainland Southeast Asia (B); the African subcontinent (C); Australia (D). For fine-scale analyses only for the peninsular Indian Plate each species was assigned based on their distribution to the following areas: northern Western Ghats (E); central Western Ghats (F); southern Western Ghats (G); and Eastern Ghats (H). For both datasets the maximum number of areas allowed was set to four. Four models were implemented in BioGeoBEARS (DIVA, DIVA+J, DEC, and DEC + J), and likelihood scores compared under AIC. In these analyses, we explored the role of three processes (dispersal, vicariance, and extinction), implemented as free parameters in a maximum likelihood framework and estimated from the data.

## Results

**Fig. 2:**
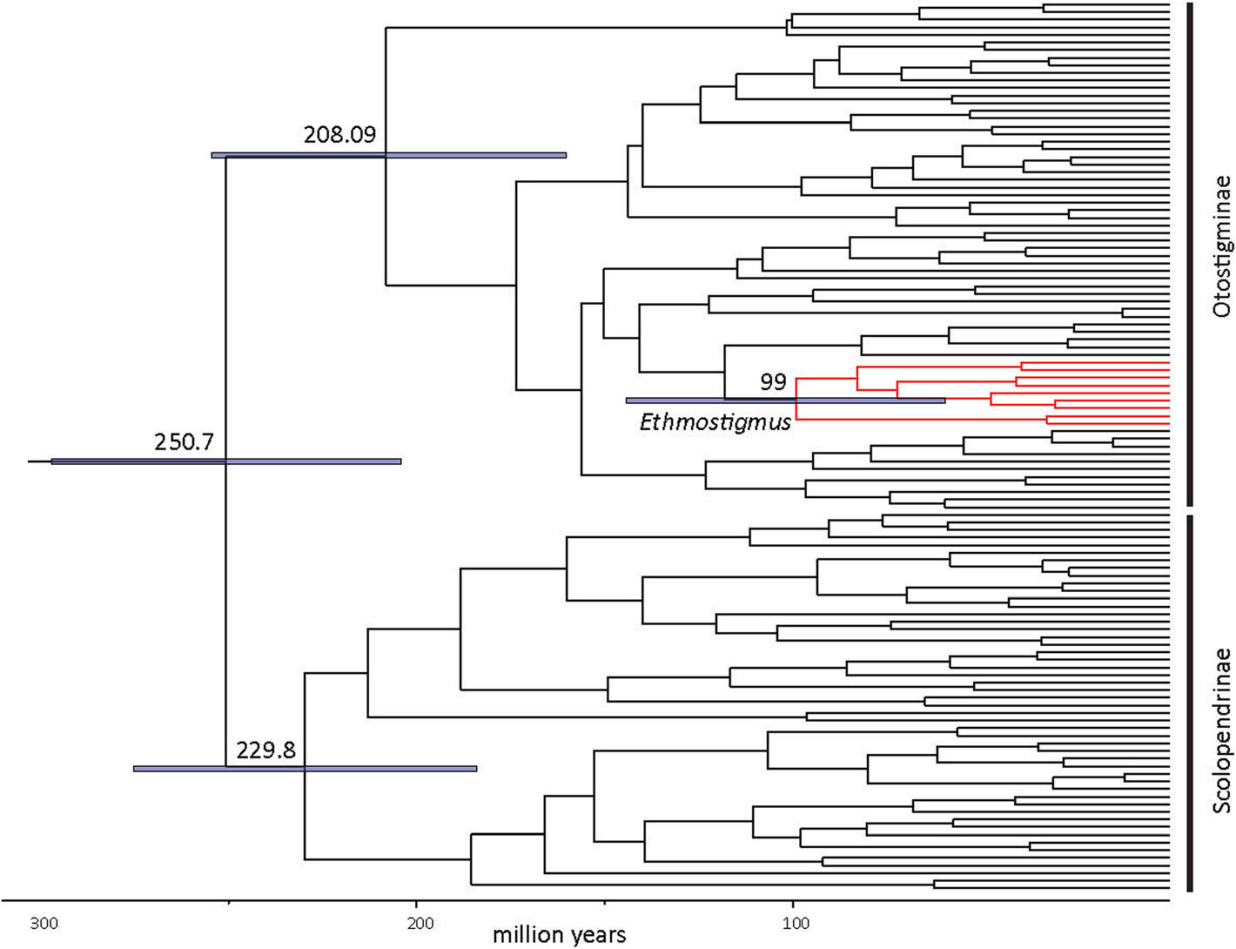
Time-tree for the family Scolopendridae, estimating diversification beginning near the Permian-Triassic boundary

**Fig. 3:**
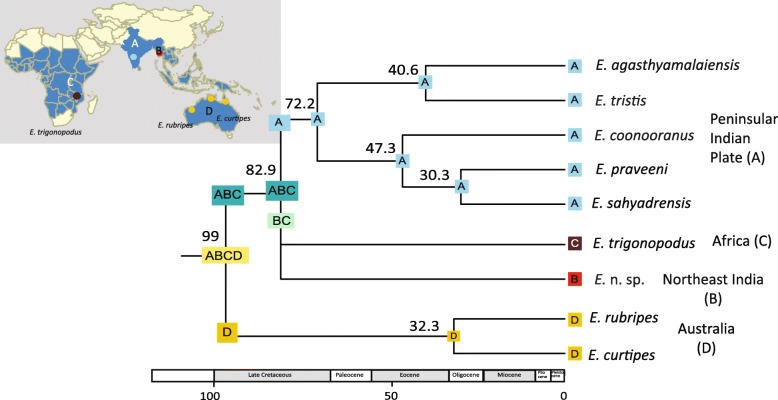
Species time-tree with ancestral areas for *Ethmostigmus* in the Old World tropics; reconstructed ancestral areas are shown at each node and vicariance events shown on the branches

The family Scolopendridae started diversifying towards the end of Permian to the Early Triassic, around 250.6 Ma (95% HPD range 303–190 Mya) (Fig. 2). The targeted group, *Ethmostigmus*, began its diversification in the Late Cretaceous, at 99 Ma (95% HPD range 150–54 Mya), when Australian species separated from other congeners. The African and North-east Indian species separated from a peninsular Indian clade in the Late Cretaceous (82.9 Ma, 95% HPD 145–45 Mya). Diversification of the peninsular Indian clade began around 72.2 Ma (95% HPD range 118–35 Mya) (Fig. 3), and species-level diversification commenced in the Cenozoic, 47.3–30.3 Mya. In historical biogeographic analyses of *Ethmostigmus*, both DEC and DIVA methods with or without the ‘j’ parameter (for founder event speciation) resulted in identical ancestral areas and likelihood scores (Table 1), and thus the results of only one of the models, DEC (LnL = − 6.51), are shown in Fig. 2. Both the methods suggested a widespread ancestor in the Old World tropics. This was followed by a vicariance event in the Late Cretaceous in which Australian species separated from peninsular Indian, North-east Indian and African species. Another vicariance event separated peninsular India from NE India and Africa (Fig. 3). Given the uncertainty in phylogenetic relationships between African (*E. trigonopodus*) and North-east Indian species (*E.* n. sp.), no inference was made on the ancestral area reconstruction for those two species (Additional file 1: Figure S1).

**Table 1:** Ancestral range reconstruction inferred in BioGeoBEARS for *Ethmostigmus* and for its PIP clade alone using two methods, dispersal–extinction–cladogenesis (DEC) and dispersal–vicariance analysis (DIVA) with a founder-event speciation (j) parameter

Models	LogLikelihood	No. of Parameters in model	AIC	AIC weight	Dispersal rate (d)	Extinction rate (e)	Jump dispersal (j-founder-event speciation)
*Ethmostigmus*							
**DEC**	**−6.51**	**2**	**17.02**	**0.1**	**1.00E-12**	**1.00E-12**	**0**
DEC + J	−6.93	3	19.85	0.035	1.00E-12	1.00E-12	0.033
DIVALIKE	−5.10	2	14.24	0.58	1.00E-12	1.00E-12	0
DIVALIKE+J	−5.12	3	16.20	0.21	1.00E-11	1.00E-12	0.008
*Ethmostigmus* peninsular Indian clade							
DEC	−11.83	2	27.66	0.0041	0.0025	0.0047	0
**DEC + J**	**−6.17**	**3**	**18.34**	**0.43**	**1.00E-12**	**1.00E-12**	**2.17**
DIVALIKE	−10.43	2	24.85	0.017	0.0018	1.00E-12	0
DIVALIKE+J	−6.06	3	18.12	0.49	1.00E-12	1.00E-12	1.43

The AIC weights compare pairs of models (e.g. DEC vs. DEC + J). The models marked in bold are discussed in the text and presented in Figs. 2 and 3, respectively

**Fig. 4:**
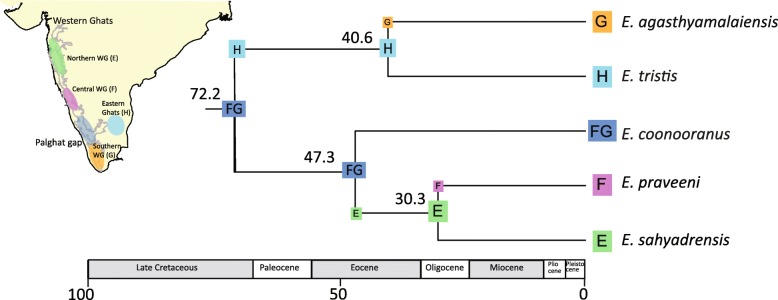
*Ethmostigmus* species time-tree for peninsular Indian taxa with ancestral areas; reconstructed ancestral areas are shown at each node and dispersal events shown on the branches

Further, we reconstructed historical biogeography of the endemic PIP clade, which consists of five species, to examine fine-scale biogeographic and diversification patterns. DEC and DIVA both yielded identical ancestral area reconstructions (Table 1), thus only DEC + j (LnL = − 6.17) is shown in Fig. 4. The southern and central Western Ghats are inferred to be the ancestral area to the PIP clade, with four independent dispersal events (Fig. 4). One of these was from the southern-central Western Ghats to the Eastern Ghats during the Late Cretaceous, making the Eastern Ghats the ancestral area for the *E. agasthyamalaiensis* and *E. tristis* clade. This was followed by a back dispersal event from the Eastern Ghats to the southern Western Ghats leading to *E. agasthyamalaiensis*. Both species diverged from each in the Eocene (40.6 Ma, 95% HPD range 80–20 Mya). Within the Western Ghats clade, the Southern-Central WG was again inferred as the ancestral area, with one dispersal event into northern parts of the Western Ghats in the Eocene (47 Ma, 95% HPD range 80–20 Mya), making northern parts of the WG the ancestral area for a clade of *E. praveeni* and *E. sahyadrensis*. There was then dispersal from northern to central parts of the Western Ghats where *E. praveeni* and *E. sahyadrensis* beginning to diverge in the Oligocene, 33.3 Ma (95% HPD range 54–10 Mya).

## Discussion

### Old World tropical biogeography

The deep divergence estimates and biogeographic analyses for *Ethmostigmus* suggest that its early evolutionary history was shaped by Gondwanan break-up. Historical biogeographic analyses suggested a widespread ancestor across East and West Gondwanan landmasses (Africa, Australia, and the Indian subcontinent). Both vicariance events that separating the peninsular Indian clade from Australian and African species occurred during the Late Cretaceous. The credible intervals around the median ages are large but nonetheless fall within the timeframe of Gondwana breakup. *Ethmostigmus* can be inferred to have a Gondwanan origin and has an in-situ radiation in peninsular India [23], as is likely the case in Australia as well [24]. Currently, we have included only one endemic species from North-east India (geographically closest to Southeast Asia). Interestingly, it was not part of the peninsular Indian clade but was part of a clade comprising of species from Australia and Africa, a trichotomy observed between these regions in Bayesian phylogenetic analyses (Additional file 1: Figure S1). Therefore, we retained the uncertainty in biogeographic analyses and refrain from commenting on the ancestral area reconstruction specifically for African and North-east Indian species. A detailed taxon sampling across Southeast Asia, Wallacea and Melanesia is needed in order to evaluate possible scenarios for species in these regions, e.g., whether their evolutionary history was influenced by dispersal/vicariance from Australasia or dispersal out of India or Africa.

To our knowledge, this is one of few studies on ancient terrestrial invertebrates using fossils to calibrate phylogenetic trees to defend Gondwanan break-up playing a significant role in shaping a clade’s biogeographic history at the scale of the Old World tropics. Examples from other groups of organisms are likewise uncommon. A study on the plant family Exaceae used calibrated molecular phylogeny to evoke Gondwanan vicariance as an explanation for disjunct distributions of the genus *Exacum* in Africa, Madagascar and the Indian subcontinent [66]. One of the main limiting factors in testing Gondwana vicariance and/or long-distance dispersal hypotheses in many studies has been incomplete taxon sampling, wherein the Indian subcontinent and/or Asian taxa are missing, or reliable fossils are unavailable to calibrate molecular phylogenies. At a more restricted geographic scale, a few studies on taxa from India-Madagascar or India-Seychelles have invoked Gondwana breakup. Among aquatic beetles of the family Hydrophilidae, a dated molecular phylogeny showed that lineages from India–Madagascar diverged in the Cretaceous, consistent with Gondwanan vicariance, and the authors compiled information from other studies in which Gondwana breakup influenced distributions, including frogs, plants, and likely caecilians [67]. In contrast, in the case of Mycalesina butterflies, a group with an Old World tropical distribution, both vicariance and dispersal events explained the biogeographic patterns, but these events happened relatively recently, in the Oligocene and Miocene [68]. Similarly, many other taxa with typical Gondwanan distributions have been attributed to dispersal because of their younger divergence time estimates [69].

### Biogeography and diversification in PIP

The divergence and biogeographic analyses suggested a Gondwanan origin of the *Ethmostigmus* clade in peninsular India. The PIP clade started diversifying in the Late Cretaceous when the peninsular Indian plate was drifting towards Asia and was mostly isolated from other landmasses. This is consistent with an earlier study on Indian Scolopendridae in which four other genera, namely *Cormocephalus*, *Digitipes, Rhysida*, and *Scolopendra* were also inferred to have Late Cretaceous origins [28]. Apart from scolopendrid centipedes, a few other taxa have been suggested to have an ancient Gondwanan history in PIP, including frogs – family Nasikabatrachidae [70]; family Nyctibatrachidae [71]; caecilians – family Indotyphlidae [41]; beetles – family Hydrophilidae; plants – *Exacum* [66]. Most of these ancient endemic lineages are restricted to the wet forests of the Western Ghats, although it has also been argued that the Eastern Ghats could also potentially harbour ancient lineages but they remain poorly explored [41, 72, 73]. In this study, we report one of the oldest divergences and lack of gene flow among the Eastern and Western Ghats wet forest endemics, between *E. agasthyamalaiensis - E. tristis*, dated to 40 Ma (95% HPD range 80–20 Mya). Another known example of ancient divergence in the Eastern and Western Ghats comes from caecilians with a Gondwanan origin (the genus *Gegeneophis*). This was dated to ca 35 Ma and attributed to pre-Miocene wet-zone fragmentation [41]. In a group of co-occurring geckos (Genus: *Geckoella*), the Eastern-Western Ghats split was shown to date to 32–24 Mya [35] and in another group of lizards (Genus: *Sarada* and *Sitana*), it was much more recently in the mid-Miocene [37]. It is noteworthy that irrespective of the evolutionary origins, either Gondwanan or Asian, Eastern and Western Ghats taxa have been distinct and lack gene flow.

The PIP clade underwent in-situ speciation, forming an endemic radiation of five species through four dispersal events throughout the Cenozoic (Fig. 4). *Ethmostigmus* occupies a wide variety of habitats, ranging from rain forests to savanna in Africa and Australia, whereas in PIP species diversified only in wet forests in both in the Western and Eastern Ghats and not in the adjacent dry forests. Among the four dispersal events inferred for the PIP radiation, one was at the supraspecific clade level and occurred in the Late Cretaceous to Paleocene interval. It was from the southern-central part of the Western Ghats to the Eastern Ghats (Fig. 4). Based on plant fossils it was postulated that western and north-western parts of PIP were re-established with wet evergreen forests in the Early Paleocene after extensive volcanic activity [32, 33]. Some recent studies also argue that the monsoon or seasonal wet climates existed throughout the Paleogene [74]. In another scolopendrid genus in the Western Ghats, *Digitipes*, dispersal events are inferred to have shaped its early evolutionary history in the Paleocene from southern to central and northern parts of the Western Ghats [32]. It was suggested that the re-establishment of the wet forests in the Paleocene may have facilitated these dispersal events, southern parts acting as a refugium during volcanic activity. One of the oldest species in the present study, *E. coonooranus*, has a relatively broad geographic distribution in the central and southern parts of the Western Ghats, its diversification commencing in the Early Eocene (ca 47.3 Ma). Additionally, two dispersal events date to the Eocene, one from the Eastern Ghats to the southern Western Ghats (ca 40.6 Ma) and another from the central-southern Western Ghats to the northern Western Ghats (ca 47.3 Ma). The Eocene was marked by the regionalization and diversification of Indian flora during the Early Eocene Climatic Optimum as well as the establishment of monsoonal seasonal climate [75, 76]. Species-level diversification of the scolopendrid *Digitipes* in the Western Ghats is dated to the Eocene [32]. Apart from centipedes, the frog genus *Nyctibatrachus* (family Nyctibatrachidae) and lizards from the genus *Hemidactylus* also diversified in the Eocene in PIP [35, 71]. The last dispersal event was in the Oligocene (ca 30 Ma), from the northern Western Ghats to the central Western Ghats. Late Eocene and Oligocene climate was marked by decreased temperatures, with pronounced cooling and drying at ca. 34 Ma [77, 78]. In a few groups, initial diversification has been noted in the Oligocene, such as bush frogs (Genus: *Raorchestes*) [79], geckos (Genus: *Geckoella)* [35], and caecilians (Genus: *Gegeneophis*) [41].

Thus, multiple palaeoclimatic events throughout the Cenozoic likely influenced the diversification of the *Ethmostigmus* species in PIP. However, the low species number (five species) does not allow assessment of the effect of any of the palaeoclimatic events in a quantitative framework (e.g., Bayesian Analysis of Macroevolutionary Mixtures -BAMM). Centipedes are carnivorous predators and low species diversity within a genus is a common phenomenon (http://chilobase.biologia.unipd.it/ [22, 80]). Similar diversity patterns may likewise occur in other carnivorous terrestrial invertebrates such as spiders and scorpions. It would be worth exploring entire assemblages of predatory terrestrial invertebrates using molecular phylogenies with divergence time estimates and then assessing their diversification patterns and underlying processes across tropical landscapes.

## Conclusions

The genus *Ethmostigmus* has a Gondwanan origin and higher-level evolutionary history of the genus was influenced by Gondwanan vicariance events (Fig. 3), whereas multiple independent dispersal events governed diversification and speciation in PIP (Fig. 4) in the Cenozoic. Comparable studies using detailed species-level molecular phylogenies with divergence dates for multiple taxa will be important in understanding the roles played by vicariance and dispersal events on evolutionary history and diversification of biotas of the Old World tropics.

## Additional files

Additional file 1: **Figure S1.** Bayesian phylogenetic tree based on combined data for *Ethmostigmus* indicating Bayesian posterior probability (PP > 0.5) and ML bootstrap support (BS > 70%) at each node. (JPG 109 kb)

Additional file 2: **Table S1.** List of centipede taxa included in the phylogenetic analyses and divergence time estimation along with GenBank accession number and geographic distribution (XLSX 43 kb)

## Abbreviations

16S rRNA: 16S ribosomal RNA
28S rRNA: 28S ribosomal RNA
AIC: Akaike Information Criterion
BAMM: Bayesian Analysis of Macroevolutionary Mixtures
BioGeoBEARS: BioGeography with Bayesian (and likelihood) Evolutionary Analysis of RangeS
COI: Cytochrome *c* oxidase subunit I
DEC: Dispersal-extinction-cladogenesis
DIVA: Dispersal-vicariance analysis
MSC: Multi-species coalescent
PIP: Peninsular Indian Plate
