# Correction to: Evolutionary biogeography of the centipede genus Ethmostigmus from Peninsular India: testing an ancient vicariance hypothesis for Old World tropical diversity

**DOI:** 10.1186/s12862-019-1383-6

**Published:** 2019-02-14

**Authors:** Jahnavi Joshi, Gregory D. Edgecombe

**Affiliations:** The Natural History Museum, Cromwell Road, London, SW7 5BD UK

**Correction to: BMC Evol Biol**

**https://doi.org/10.1186/s12862-019-1367-6**

Following publication of the original article [1], the authors notified us of an error in the Results section of the Abstract. The original article has been corrected.

- Originally, the first phrase of the Results section was published as:

Divergence time estimates suggest that Ethmostigmus began diversifying in the **Early** Cretaceous, **125.4** (± 25) million years ago (Ma), its early biogeographic history shaped by vicariance. Members of Ethmostigmus in PIP form a monophyletic group that underwent endemic radiation in the Late Cretaceous, **100** (± 25) Ma.

- The phrase was corrected to:

Divergence time estimates suggest that Ethmostigmus began diversifying in the **Late** Cretaceous, **99** (± 25) million years ago (Ma), its early biogeographic history shaped by vicariance. Members of Ethmostigmus in PIP form a monophyletic group that underwent endemic radiation in the Late Cretaceous, **72** (± 25) Ma.

## References

[1] Joshi J, Edgecombe GD (2019). Evolutionary biogeography of the centipede genus Ethmostigmus from Peninsular India: testing an ancient vicariance hypothesis for Old World tropical diversity. BMC Evol Biol.

