# Supplementary figures and images for: Evolutionary biogeography of the centipede genus Ethmostigmus from Peninsular India: testing an ancient vicariance hypothesis for Old World tropical diversity
Source: BMC Evol Biol. 2019 Feb 1;19:41. doi: 10.1186/s12862-019-1367-6 (PMC6359765; doi:10.1186/s12862-019-1367-6)

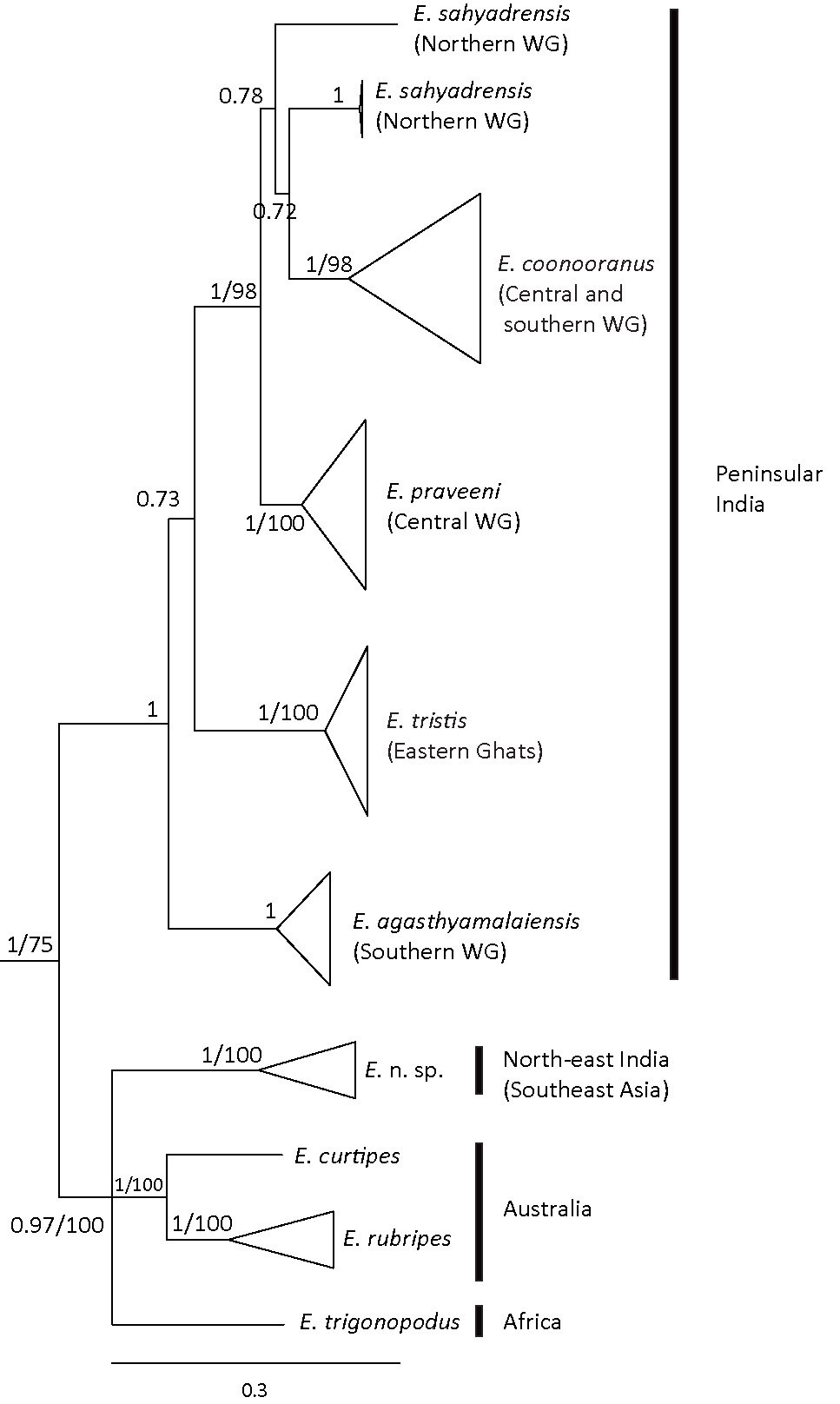

Supplement: Supplementary file 1 — Figure S1. Bayesian phylogenetic tree based on combined data for Ethmostigmus indicating Bayesian posterior probability (PP > 0.5) and ML bootstrap support (BS > 70%) at each node. (JPG 109 kb) [file 12862_2019_1367_MOESM1_ESM.jpg]
